# Optimal Geometry and Motion Coordination for Multisensor Target Tracking with Bearings-Only Measurements

**DOI:** 10.3390/s23146408

**Published:** 2023-07-14

**Authors:** Shen Wang, Yinya Li, Guoqing Qi, Andong Sheng

**Affiliations:** School of Automation, Nanjing University of Science and Technology, Nanjing 210094, China

**Keywords:** optimal geometry, bearings-only measurement, Fisher information matrix, motion coordination

## Abstract

This paper focuses on the optimal geometry and motion coordination problem of mobile bearings-only sensors for improving target tracking performance. A general optimal sensor–target geometry is derived with uniform sensor–target distance using D-optimality for arbitrary *n* (n≥2) bearings-only sensors. The optimal geometry is characterized by the partition cases dividing *n* into the sum of integers no less than two. Then, a motion coordination method is developed to steer the sensors to reach the circular radius orbit (CRO) around the target with a minimum sensor–target distance and move with a circular formation. The sensors are first driven to approach the target directly when outside the CRO. When the sensor reaches the CRO, they are then allocated to different subsets according to the partition cases through matching the optimal geometry. The sensor motion is optimized under constraints to achieve the matched optimal geometry by minimizing the sum of the distance traveled by the sensors. Finally, two illustrative examples are used to demonstrate the effectiveness of the proposed approach.

## 1. Introduction

Bearings-only target tracking is widely applied in wireless sensor networks for the civilian and military areas [1,2]. Different from other sensors such as range-only sensors, time difference of arrival (TDOA) sensors, and so on, bearings-only sensors work in passive mode and easily survive from being detected and attacked. However, they are highly sensitive to a range in which even a small angle measurement error may lead to a large tracking error. Therefore, bearings-only target tracking has been a research area of considerable interest for decades. Meanwhile, with the development of the unmanned vehicles, the traditional stationary sensor platforms have evolved into mobile ones characterized by high speed and long endurance. Accordingly, flexible sensor motion coordination can be achieved, so the tracking accuracy and the survival ability are significantly improved from sensor coordination.

Much previous work has been dedicated to developing different estimators for target tracking based on bearings-only measurements in two- and three-dimensional space [3,4,5,6]. The extended Kalman filter (EKF) is a classical method for the nonlinear tracking problem [7] but often diverges when the model nonlinearity is strong. The pseudolinear Kalman filter (PLKF) was introduced in [8,9], with better convergence than the EKF. However, the estimate is biased, which is highly dependent on sensor geometry [10]. Furthermore, other estimation algorithms such as the unscented Kalman filter (UKF) [11], cubature Kalman filter (CKF) [12,13,14,15], and particle filter (PF) [16,17] have been applied in bearings-only target tracking with different estimation performance advantages.

Compared with the improvement in the tracking accuracy produced by the estimation algorithms, sensor–target geometry plays a fundamental role in determining the accuracy of target tracking systems [18,19,20,21]. The Fisher information matrix (FIM) is a commonly used criterion assessing target tracking accuracy. The inverse of the FIM, called the Cramer–Rao lower bound (CRLB), indicates the optimal performance of a tracking system. Three popular optimality criteria are adopted tp achieve the optimal sensor configuration based on the FIM [19]. D-optimality minimizes the area of the uncertainty ellipse by maximizing the determinant of the FIM [18,20,22]; A-optimality suppresses the average variance by minimizing the trace of CRLB [23,24]; and E-optimality minimizes the length of the largest axis of the same ellipsoid by minimizing the maximum eigenvalue of the CRLB [19]. In [25], D-optimality was adopted to optimize sensor placement for range-based target tracking. In [26], the conditions for optimal placement of heterogeneous sensors were derived based on maximizing the information matrix, and the optimal placement for paired sensors was developed leveraging a “divide-and-conquer” strategy. In [27], A-optimality was used to solve sensor placement for 3D angle-of-arriving target localization. Geometric dilution of precision (GDOP) [28] is another criterion used to evaluate tracking accuracy. GDOP is defined as the root mean square position error and illustrates how an estimation is influenced by sensor–target geometry [29]. The optimal deployment for multitarget localization was developed in [30] by minimizing the GDOP.

In addition to the above theoretical analysis on the sensor–target geometries, some sensor path optimization methods have been proposed for target tracking to avoid the difficulty in finding the closed-form solution. A gradient-descent-based motion planning algorithm was presented for decentralized target tracking [31]. In [32], a gradient descent optimization algorithm was proposed for single- and multisensor path planning by minimizing the mean square error in 2D space. In [33], the path optimization for passive emitter localization in 2D space was transformed into a nonlinear programming problem with the FIM as the cost function. In [34], the path optimization strategy for 3D AOA target tracking was developed by minimizing the trace of covariance matrices with gradient descent optimization and a grid search method. In [35], the optimal sensor placement for AOA sensors was derived with a Gaussian prior using D- and A-optimality. In addition, the result was extended to path optimization based on a projection algorithm.

Most of the existing work has focused on optimal deployment using multiple bearings-only sensors for target localization. Some closed-form solutions have been derived with equal angular distribution. Inspired by the “divide-and-conquer” strategy in [26], the continuum of the optimal solution for bearings-only measurement has potential to be extended to general circumstances. Moreover, for bearings-only target tracking problems using mobile sensors, some studies in the literature have adopted optimization methods such as gradient descent, Gauss–Seidel relaxation, and so on. Nevertheless, the solution space for optimization is complex due to the high nonlinearity of the cost functions related to the FIM. As a result, these numerical methods may lead to falling into local optima and fail to reach the globally optimal tracking performance. Motivated by the aforementioned aspects, this paper focuses on the optimal sensor–target geometry and motion coordination problem of mobile bearings-only sensors for target tracking. The sensors are driven to approach the target from a distance and eventually move in a circular formation to track the target.

The contributions of this paper are summarized as follows. (1) The suboptimality of approaching the target for bearings-only sensors to improve tracking performance is analyzed. (2) A continuum solution to optimal sensor–target geometry is derived with uniform sensor–target distance using D-optimality for arbitrary *n* (n≥2) bearings-only sensors. The optimal geometry is characterized by the partition cases dividing *n* into the sum of integers no less than two. (3) A motion coordination algorithm to achieve globally optimal performance is developed based on matching optimal geometry and motion optimization to achieve the optimal target tracking performance.

The remainder of this paper is organized as follows: Section 2 presents the problem formulation. The CKF and FIM are introduced in Section 3. Section 4 reformulates the problem and investigates the optimality analysis. In Section 5, we design a motion coordination strategy based on the results in Section 4. The proposed method is verified by simulations in Section 6. Section 7 concludes this paper.

Notations: Define $\theta_{i}\in \left(-\pi ,\pi \right]$, $\theta_{ij}=\theta_{j}-\theta_{i}\;\in \left(-\pi ,\pi \right]$. The two-norm of a vector $x\in R^{n}$ is defined as $∥x∥=\sqrt{x^{T}x}$. Chol(M) indicates the Cholesky decomposition of M. tr(·) and det(·) denote the trace and the determinant of the matrix contained in the bracket, respectively. |S| represents the cardinality of the set *S*. S\T={e|e∈Sande∉T}.

## 2. Problem Formulation

**Figure 1:**
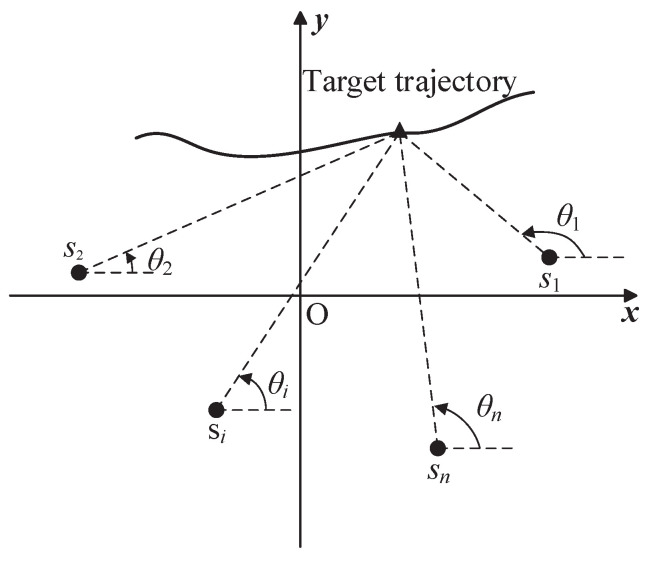
Target tracking geometry for n>2 bearings-only sensors, where i>2.

This paper focuses on the problem of sensor motion and coordination for single-moving-target tracking with n≥2 bearings-only sensors in 2D space. The target tracking geometry is depicted in Figure 1. $\theta_{i,k}$ is the angle of the line of sight (LOS) from sensor *i* at discrete time *k*. Define $z_{i,k}$ as the measurement of $\theta_{i,k}$; then, the measurement function is

(1) $$z_{i,k}=\theta_{i,k}+\eta_{i,k}=\tan^{-1}\left(\frac{y_{k}^{p}-y_{i}\left(k\right)}{x_{k}^{p}-x_{i}\left(k\right)}\right)+\eta_{i,k}$$

where $p_{k}={\left[x_{k}^{p},y_{k}^{p}\right]}^{T}$ is the position of the target at time *k*; $\tan^{-1}\left(\cdot \right)$ is the four-quadrant inverse tangent function and $\theta_{i,k}\in \left(-\pi ,\pi \right]$; $s_{i}\left(k\right)={\left[x_{i}\left(k\right),y_{i}\left(k\right)\right]}^{T}$ is the location of sensor *i*; $\eta_{i,k}$ is the measurement noise and assumed to be i.i.d Gaussian noise with zero mean and variance $\sigma_{i}^{2}$, i∈{1,2,…,n}. The sensors are homogeneous, i.e., $\sigma_{i}^{2}=\sigma_{\theta }^{2}$. Write the measurements in a compact form as $z_{k}={\left[z_{1,k},z_{2,k},\ldots ,z_{n,k}\right]}^{T}\in R^{n}$, and $\eta_{k}={\left[\eta_{1,k},\eta_{2,k},\ldots ,\eta_{n,k}\right]}^{T}\in R^{n}$ is measurement Gaussian noise with zero mean and covariance $R_{k}=\sigma_{\theta }^{2}I$, where I is an identity matrix.

Consider the target whose motion is described by a nonlinear dynamic discrete system

(2) $$x_{k+1}=f\left(x_{k}\right)+w_{k}$$

where $x_{k}\in R^{n_{x}}$ is the state vector of the dynamic system at discrete time *k*; $w_{k}\in R^{n_{x}}$ is process Gaussian noise with zero mean and covariance $Q_{k}$; $n_{x}$ is the dimension of the state vector. Meanwhile, $w_{k}$ and $\eta_{k}$ are mutually independent processes.

The dynamic model of the mobile sensors is given by

(3) $$\begin{array}{cc} s_{i}\left(k+1\right) & =s_{i}\left(k\right)+u_{i}\left(k\right)\\ u_{i}\left(k\right) & =v_{i}\left(k\right)T \end{array}$$

where $s_{i}\left(k\right)$ is the position of sensor *i* at discrete time *k*; $u_{i}\left(k\right)$ is the control input for sensor *i* at time *k*; $v_{i}\left(k\right)$ is the designed velocity of sensor *i* at time *k*; and *T* is the sampling time.

The state parameters of the target are unknown. We assume that the state of the mobile sensors and the measurements taken by them are known. Because of the noncooperative scenario, the minimum distance restriction between the target and the sensors should be ensured. We aimed to estimate the target state using the bearings-only measurements and improve the tracking accuracy by optimizing the sensor–target geometry of cooperative mobile sensors under practical constraints.

**Assumption A1.**  *At the beginning of the tracking process, at least two sensors are deployed in positions that are not colinear to the target to ensure the observability of the target by the sensors [19,36].*

**Assumption** **A2.**  *The mobile sensors are homogeneous with a maximum speed $v_{\max}$ and a maximum turn rate $\varphi_{\max}$ due to the limitations of the mechanical properties. The maximum speed of the sensor is faster than that of the target to ensure they can catch up the target. The minimum distance between sensors and the target is denoted as $d_{\min}$.*

## 3. Parameter Estimation

In this paper, we use the cubature Kalman filter [12] to estimate the state of the target. The CKF is a nonlinear filter rising in the past decade with improved performance over the conventional nonlinear filter, particularly in addressing the strong nonlinearity in bearings-only target tracking.

In addition, it is known that the tracking performance of static sensors has a limited track range performance. Obviously, one feasible way to improve the tracking accuracy is moving sensors to better locations to accurately track the target. Therefore, the FIM based on bearings-only measurements is introduced in this section for optimality analysis in the following section.

### 3.1. Cubature Kalman Filter

Denote ${\hat{x}}_{k|k}$ as the estimate of $x_{k}$ and $P_{k|k}$ as the estimate error covariance by using the bearings-only measurements $z_{k}$. The cubature Kalman filter, in its time- and measurement-update forms, can be computed by starting from ${\hat{x}}_{0|0}$ and $P_{0|0}$. The iteration functions are as follows:

Step 1. Evaluate cubature points ($i=1,2,\ldots ,2n_{x}$)

(4) $$\begin{array}{cc} S_{k-1|k-1} & =\mathrm{Chol}\left(P_{k-1|k-1}\right)\\ X_{i,k-1|k-1} & =S_{k-1|k-1}\xi_{i}+{\hat{x}}_{k-1|k-1} \end{array}$$

where $S_{k-1|k-1}$ is the Cholesky decomposition of $P_{k-1|k-1}$; $\xi_{i}=\sqrt{n_{x}}{\left[1\right]}_{i}$; ${\left[1\right]}_{i}\in R^{n_{x}}$ represents the *i*th element of the following set

$$\underset{2n_{x}}{\underset{︸}{\left\{\left[\begin{array}{c} 1\\ 0\\ \vdots \\ 0 \end{array}\right],\left[\begin{array}{c} 0\\ 1\\ \vdots \\ 0 \end{array}\right],\ldots ,\left[\begin{array}{c} 0\\ 0\\ \vdots \\ 1 \end{array}\right],\left[\begin{array}{c} -1\\ 0\\ \vdots \\ 0 \end{array}\right],\left[\begin{array}{c} 0\\ -1\\ \vdots \\ 0 \end{array}\right],\ldots ,\left[\begin{array}{c} 0\\ 0\\ \vdots \\ -1 \end{array}\right]\right\}}}$$

Step 2. Time update

(5) $$\begin{array}{cc} X_{i,k|k-1} & =f(X_{i,k-1|k-1})\\ {\hat{x}}_{k|k-1} & =\frac{1}{2n_{x}}\sum_{i=1}^{2n_{x}}X_{i,k|k-1}\\ P_{k|k-1} & =\frac{1}{2n_{x}}\sum_{i=1}^{2n_{x}}X_{i,k|k-1}X_{i,k|k-1}^{T}-{\hat{x}}_{k|k-1}{\hat{x}}_{k|k-1}^{T}+Q_{k-1} \end{array}$$

where ${\hat{x}}_{k|k-1}$ is the state prediction, and $P_{k|k-1}$ is the predicted error covariance.

Step 3. Measurement update

(6) $$\begin{array}{cc} S_{k|k-1} & =\mathrm{Chol}\left(P_{k|k-1}\right)\\ \chi_{i,k|k-1} & =S_{k|k-1}\xi_{i}+{\hat{x}}_{k|k-1}\\ Z_{i,k|k-1} & =h(\chi_{i,k|k-1})\\ {\hat{z}}_{k|k-1} & =\frac{1}{2n_{x}}\sum_{i=1}^{2n_{x}}Z_{i,k|k-1}\\ P_{zz,k|k-1} & =\frac{1}{2n_{x}}\sum_{i=1}^{2n_{x}}Z_{i,k|k-1}Z_{i,k|k-1}^{T}-{\hat{z}}_{k|k-1}{\hat{z}}_{k|k-1}^{T}+R_{k}\\ P_{xz,k|k-1} & =\frac{1}{2n_{x}}\sum_{i=1}^{2n_{x}}\chi_{i,k|k-1}Z_{i,k|k-1}^{T}-{\hat{x}}_{k|k-1}{\hat{z}}_{k|k-1}^{T}\\ W_{k} & =P_{xz,k|k-1}P_{zz,k|k-1}^{-1}\\ {\hat{x}}_{k|k} & ={\hat{x}}_{k|k-1}+W_{k}\left(z_{k}-{\hat{z}}_{k|k-1}\right)\\ P_{k|k} & =P_{k|k-1}-W_{k}P_{zz,k|k-1}W_{k}^{T} \end{array}$$

where ${\hat{z}}_{k|k-1}$ is the predicted measurement; $P_{zz,k|k-1}$ is the innovation covariance matrix; $P_{xz,k|k-1}$ is the cross-covariance matrix; $W_{k}$ is the Kalman gain.

### 3.2. Fisher Information Matrix

The error covariance matrix is defined as

(7) $$P_{k|k}≜E\left[\left(x_{k}-{\hat{x}}_{k|k}\right){\left(x_{k}-{\hat{x}}_{k|k}\right)}^{T}\right]\geq J_{k}^{-1}$$

where $J_{k}$ is called the FIM, which quantifies the amount of information obtained from the measurements, with the expression

(8) $$J_{k}=E\left[-\frac{\partial^{2}\ln p\left(z_{k}|x_{k}\right)}{\partial x_{k}^{2}}\right]$$

where $p(z_{k}|x_{k})$ is the probability density function, expressed as

(9) $$p\left(z_{k}|x_{k}\right)=\frac{1}{\sqrt{{\left(2\pi \right)}^{n}\det\left(R_{k}\right)}}\times \exp\left\{-\frac{1}{2}[{\left(z_{k}-h\left(x_{k}\right)\right]}^{T}R_{k}^{-1}[\left(z_{k}-h\left(x_{k}\right)\right]\right\}$$

Given the measurements vector $z_{k}$, the FIM is determined as

(10) $$J_{k}=\frac{1}{\sigma_{\theta }^{2}}\sum_{i=1}^{n}\frac{1}{r_{i,k}^{2}}\left[\begin{array}{cc} \cos^{2}\left(\theta_{i,k}\right) & -\frac{1}{2}\sin\left(2\theta_{i,k}\right)\\ -\frac{1}{2}\sin\left(2\theta_{i,k}\right) & \sin^{2}\left(\theta_{i,k}\right) \end{array}\right]$$

where $r_{i,k}=∥p_{k}-s_{i}\left(k\right)∥$ represents the distance between the target position $p_{k}$ and the sensor position $s_{i}\left(k\right)$ at time *k*.

**Lemma 1**  ([18]). *The FIM is expressed in *(10)*; then, the following expressions of the determinant of the FIM are equivalent:*

(11) $$\begin{array}{cc} \left(1\right)\det\left(J_{k}\right) & =\frac{1}{4\sigma_{\theta }^{4}}\left[{\left(\sum_{i=1}^{n}\frac{1}{r_{i,k}^{2}}\right)}^{2}-{\left(\sum_{i=1}^{n}\frac{\cos(2\theta_{i,k})}{r_{i,k}^{2}}\right)}^{2}-{\left(\sum_{i=1}^{N}\frac{\sin(2\theta_{i,k})}{r_{i,k}^{2}}\right)}^{2}\right]\\ \left(2\right)\det\left(J_{k}\right) & =\frac{1}{\sigma_{\theta }^{4}}\sum_{\Psi }\frac{\sin^{2}\left(\theta_{ij}\right)}{r_{i,k}^{2}r_{j,k}^{2}} \end{array}$$

*where Ψ={{i,j}} is the set of all combinations of i and j with 1≤i<j≤n; $\theta_{ij}=\theta_{j,k}-\theta_{i,k}$.*

## 4. Optimality Analysis

The problem of path planning and motion coordination for improving tracking performance is equivalent to finding the next waypoints at each time step by maximizing the determinant of the FIM. There exist two kinds of parameters influencing the determinant of the FIM. So, we can maximize $\det(J_{k})$ by simultaneously reducing the distances between the sensors and target and configuring the angles among the sensors.

In order to ensure the minimum distance constraint, the sensors move on a circular trajectory at a distance radius around the target. Before that, the path to reaching the circular radius orbit (CRO) for improving the tracking accuracy was studied. Thus, the design of the motion coordination for multiple sensors is divided into two stages, including outside the CRO distance and on the CRO distance $d_{\min}$.

### 4.1. Outside the CRO Distance

Consider the bearings-only tracking problem. When the range between the target and the sensor is greater than $d_{\min}$, the problem of the optimal sensor movement is equivalent to the following optimization problem:

(12) $$\begin{array}{cc} \max & \;\det(J_{k+1})\\ s.t. & \;∥v_{i}\left(k\right)∥\leq v_{\max}\\ \; & \;|∠v_{i}\left(k\right)-∠v_{i}\left(k-1\right)|\leq \varphi_{\max} \end{array}$$

where $∠v_{i}\left(k\right)$ is the angle of the velocity vector at time *k*, and the difference between $∠v_{i}\left(k\right)$ and $∠v_{i}\left(k-1\right)$ is bounded by $\varphi_{\max}$ due to the limited turn rate.

Obviously, the difficulty of solving problem (12) increases with the increase in the number of the mobile sensors, though they can be solved via numerical methods. As such, we turned to suboptimal motion to reduce computational complexity.

**Figure 2:**
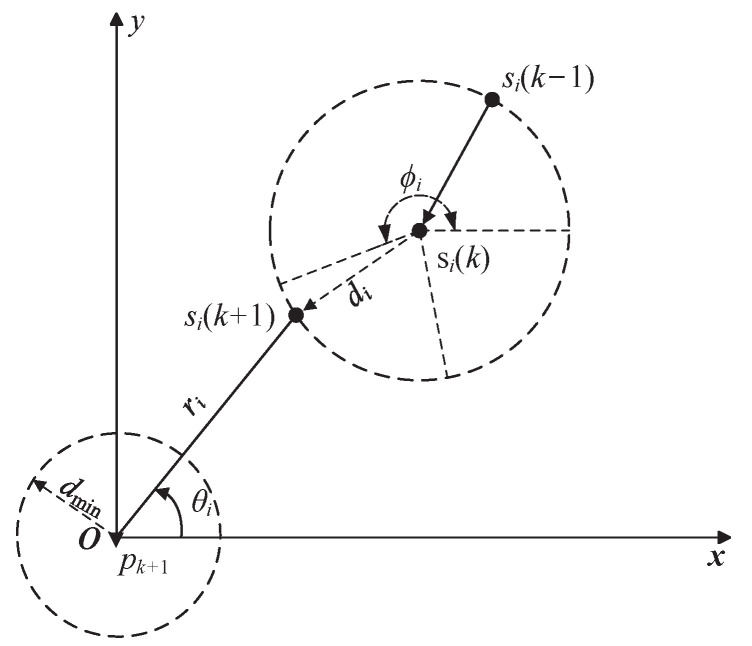
Optimal sensor motion for target tracking.

When the sensors are far away from the target, the sensors are expected to move with maximum speed $v_{\max}$ to approach the target. As shown in Figure 2, the location $s_{i}\left(k+1\right)$ that sensor *i* is able to reach can be expressed by

(13) $$\begin{array}{cc} x_{i}\left(k+1\right) & =x_{i}\left(k\right)+v_{\max}T\cos\phi_{i,k}\\ y_{i}\left(k+1\right) & =y_{i}\left(k\right)+v_{\max}T\sin\phi_{i,k} \end{array}$$

where $\phi_{i,k}\in \left[0,\;2\pi \right)$ is the heading direction of sensor *i* at time *k*. For convenience, denote $\Delta x_{i}≜x_{k+1}^{p}-x_{i}\left(k\right)$, $\Delta y_{i}≜y_{k+1}^{p}-y_{i}\left(k\right)$ and $d_{i}≜v_{\max}T$.

**Theorem 1.**  *Consider the bearings-only tracking problem. When the range between the target and the sensor is greater than $d_{\min}$, and the position of the target is $p_{k+1}$ at time k+1, the suboptimal heading direction of sensor i at time k is*

(14) $$\phi_{i,k}^{*}=\tan^{-1}\left(\frac{\Delta y_{i}}{\Delta x_{i}}\right)$$

**Proof.**  According to the Cauchy inequality,

(15) $$\begin{array}{cc} \det(J_{k+1}) & \geq \frac{1}{4\sigma_{\theta }^{4}}\left[{\left(\sum_{i=1}^{n}\frac{1}{r_{i,k+1}^{2}}\right)}^{2}-\sum_{i=1}^{n}\frac{\cos^{2}2\theta_{i,k+1}}{r_{i,k+1}^{4}}-\sum_{i=1}^{n}\frac{\sin^{2}2\theta_{i,k+1}}{r_{i,k+1}^{4}}\right]\\ \; & =\frac{1}{4\sigma_{\theta }^{4}}\left[{\left(\sum_{i=1}^{n}\frac{1}{r_{i,k+1}^{2}}\right)}^{2}-\sum_{i=1}^{n}\frac{1}{r_{i,k+1}^{4}}\right]≜F\left(\gamma \right) \end{array}$$

Consider the function

(16) $$F\left(\gamma \right)=\frac{1}{4\sigma_{\theta }^{4}}\left[{\left(\sum_{i=1}^{n}\frac{1}{r_{i,k+1}^{2}}\right)}^{2}-\sum_{i=1}^{N}\frac{1}{r_{i,k+1}^{4}}\right]$$

where $\gamma ={\left[r_{1,k+1},r_{2,k+1},\ldots ,r_{n,k+1}\right]}^{T}$.

To achieve the maximum of F(γ), take the partial derivatives of F(γ) with respect to $\phi_{i,k}$. Then, we have

(17) $$\frac{\partial F(\gamma )}{\partial \phi_{i,k}}=\frac{1}{4\sigma_{\theta }^{4}}\left[\sum_{j=1}^{n}\frac{1}{r_{j,k+1}^{2}}\cdot \frac{4d_{i}}{r_{i,k+1}^{4}}\left(\Delta y_{i}\cos\phi_{i,k}-\Delta x_{i}\sin\phi_{i,k}\right)+\frac{d_{i}}{r_{i,k+1}^{6}}\left(\Delta y_{i}\cos\phi_{i,k}-\Delta x_{i}\sin\phi_{i,k}\right)\right]$$

Let $\frac{\partial F(\gamma )}{\partial \phi_{i,k}}=0$, we obtain

(18) $$\phi^{0}=\left[\begin{array}{cccc} \tan^{-1}\left(\frac{\Delta y_{1}}{\Delta x_{1}}\right) & \tan^{-1}\left(\frac{\Delta y_{2}}{\Delta x_{2}}\right) & \cdots & \tan^{-1}\left(\frac{\Delta y_{n}}{\Delta x_{n}}\right) \end{array}\right]$$

Additionally, let $H\in R^{n\times n}$ denote the Hessian matrix of F(γ) at $\phi^{0}$, with elements

(19) $$H_{ij}=\frac{\partial^{2}F\left(\gamma \right)}{\partial \phi_{i,k}\partial \phi_{j,k}}$$

We obtain

(20) $$H_{ij}{|}_{\phi^{0}}=\left\{\begin{array}{cc} 0 & i\neq j\\ -\frac{d_{i}\Delta r_{i}}{\sigma_{\theta }^{4}r_{i,k+1}^{4}}\sum_{l=1}^{n}\frac{1}{r_{l,k+1}^{2}} & i=j \end{array}\right.$$

where $\Delta r_{i}=\sqrt{\Delta x_{i}^{2}+\Delta y_{i}^{2}}$. Obviously, H is a negative definite matrix, and as a consequence, $\phi^{0}$ is the maximum point.    □

Furthermore, taking the limitation of the turn rate into consideration, the heading direction of sensor *i* at time *k* is

(21) $$\phi_{i,k}=\left\{\begin{array}{cc} {\underline{\phi }}_{i,k} & \phi_{i,k}^{*}<{\underline{\phi }}_{i,k}\\ \phi_{i,k}^{*} & {\underline{\phi }}_{i,k}\leq \phi_{i,k}^{*}\leq {\bar{\phi }}_{i,k}\\ {\bar{\phi }}_{i,k} & \phi_{i,k}^{*}>{\bar{\phi }}_{i,k} \end{array}\right.$$

where ${\bar{\phi }}_{i,k}=∠v_{i}\left(k-1\right)+\varphi_{\max}$ and ${\underline{\phi }}_{i,k}=∠v_{i}\left(k-1\right)-\varphi_{\max}$.

Note that the determinant of FIM increases with the range between the sensors and target when the angles among the sensors remain unchanged. In other words, the optimal heading direction is always toward the target, so we can force the sensors to directly approach the CRO around the target. The tracking accuracy is improved as well but does not reach the optimum.

### 4.2. On the CRO Distance $d_{\min}$

**Figure 3:**
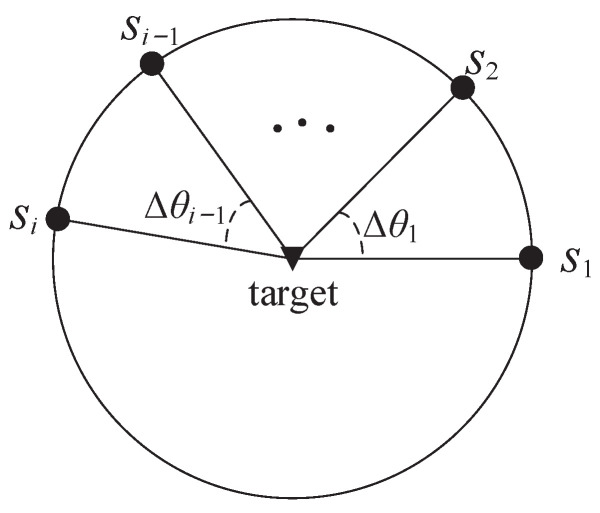
Sensor–target geometry.

When all sensors reach the CRO around the target, which is a circle centered on the target and with a radius of $d_{\min}$, we have $r_{i}=d_{\min}$. Define $\Delta \theta_{i}=\theta_{i+1}-\theta_{i}$, i∈{1,2,…,n−1}. The sensor–target geometry is depicted in Figure 3. In this section, the time step *k* is omitted for the convenience of description.

In order to simplify the analysis of optimal sensor–target geometry, the related propositions are reclaimed.

**Proposition 1.**  *The determinant of the FIM in *(11)* remains unchanged in the following three operations:*

*1.*  *Switching the position of any two sensors;*
*2.*  *Rotating all the sensors around the target;*
*3.*  *Flipping arbitrary sensors about the target.*

**Remark 1.**  *Proposition 1 originated from [18] and recognized in [20]. It implies that det(J) is invariant to these geometric operations.*

Without loss of generality, the sensors are assumed to be renumbered counterclockwise with $\theta_{i},\theta_{i}\in \left(0,\pi \right]$ through the geometric operations according to Proposition 1, which is equivalent to flipping the sensors with the actual angles of a LOS ranging from −π to 0.

The target tracking system achieves optimal estimation performance when all sensors move at the same speed as the target on the CRO in the formation, confirming the following results.

**Lemma 2**  ([30]). *Consider n bearings-only sensors tracking a single target. When all sensors are on the CRO around the target ($r_{i}=d_{\min}$), $\Delta \theta_{1}=\Delta \theta_{2}=\cdots =\Delta \theta_{n-1}=\Delta \theta$, the Fisher information determinant given in *(11)* has the upper bound $\frac{N^{2}}{4\sigma_{\theta }^{4}d_{\min}^{4}}$. The upper bound is achieved when $\Delta \theta_{i}=\frac{\pi }{n}$.*

**Remark 2.**  *When n≥3, there are two solutions for optimal geometry with equal angular distribution in [30], i.e., $\Delta \theta_{i}=\frac{\pi }{n}\;\mathrm{or}\;\frac{2}{n}\pi$. However, the optimal geometry when $\Delta \theta_{i}=\frac{2}{n}\pi$ can be obtained through flipping part of the sensors about the target in the optimal geometry when $\Delta \theta_{i}=\frac{\pi }{n}$. Therefore, we consider them as identical optimal geometry for n sensors and retain the solution of $\Delta \theta_{i}=\frac{\pi }{n}$, which avoids the complexity arising from two optional solutions.*

For a more general circumstances, there is less limitation to $\Delta \theta_{i}$. Denote S={1,2,…,n} as the set of all sensors and $S_{i}=\left\{n_{1}^{i},n_{2}^{i},\ldots ,n_{q_{i}}^{i}\right\}$ as the subset of *S*, i∈{1,2,…,m}. Denote $\Xi =\{q_{1},q_{2},\ldots ,q_{m}\}$, where $q_{i}=\left|S_{i}\right|$. Then, we have the following result:

**Theorem 2.**  *Consider the bearings-only tracking problem. When all sensors are on the CRO around the target ($r_{i}=d_{\min}$), the Fisher information determinant given in *(11)* has the upper bound $\frac{N^{2}}{4\sigma_{\theta }^{4}d_{\min}^{4}}$. The upper bound is achieved if the following conditions hold true*

(22) $$\begin{array}{cc} \overset{m}{\underset{i=1}{⋃}}S_{i}= & S,S_{i}\cap S_{j}=\emptyset \;\left(i\neq j\right)\\ \; & 2\leq q_{i}\leq n\\ \theta_{n_{l+1}^{i}}-\theta_{n_{1}^{i}}= & \frac{l}{q_{i}}\pi ,\;\forall l\in \left\{1,2,\ldots ,q_{i}-1\right\} \end{array}$$

**Proof.**  When $r_{i}=d_{\min}$, then

(23) $$\begin{array}{cc} \det(J) & =\frac{1}{\sigma_{\theta }^{4}d_{\min}^{4}}\sum_{\Psi }\sin^{2}\left(\theta_{ij}\right)\\ \; & =\frac{1}{2\sigma_{\theta }^{4}d_{\min}^{4}}\left(\frac{n(n-1)}{2}-\sum_{\Psi }\cos\left(2\theta_{ij}\right)\right) \end{array}$$

The sensors in $S_{i}$ are placed as Lemma 2. Then,

(24) $$\sum_{\Psi_{i}}^{}\cos2\left(\theta_{ab}\right)=-\frac{q_{i}}{2}$$

where $\Psi_{i}=\left\{\left\{a,b\right\}\right\}$ is the set of all combinations of *a* and *b* with a<b and $a,b\in S_{i}$. Since $\sum_{i=1}^{n}\cos\left(\alpha +\frac{2(i-1)}{n}\pi \right)=0$ (α is arbitrary, n≥2), for $j\in S_{i},\forall l\in S_{g}\left(i\neq g\right)$

(25) $$\sum_{l=1}^{q_{g}}\cos\left(2\theta_{jl}\right)=\sum_{l=1}^{q_{g}}\cos\left(2\theta_{jn_{1}^{g}}+2\left(l-1\right)\frac{\pi }{q_{v}}\right)=0$$

Finally, consider the following function

(26) $$\begin{array}{cc} \sum_{\Psi }\cos\left(2\theta_{ij}\right) & =\sum_{\Psi^{\prime }}\cos\left(2\theta_{ij}\right)+\sum_{\Psi \backslash \Psi^{\prime }}\cos\left(2\theta_{ij}\right)\\ \; & =\sum_{i=1}^{m}-\frac{q_{i}}{2}+0=-\frac{n}{2} \end{array}$$

where $\Psi^{\prime }={⋃}_{i=1}^{m}\Psi_{i}$.

Hence,

(27) $$\det\left(J\right)=\frac{n^{2}}{4\sigma_{\theta }^{4}d_{\min}^{4}}$$

   □

**Figure 4:**
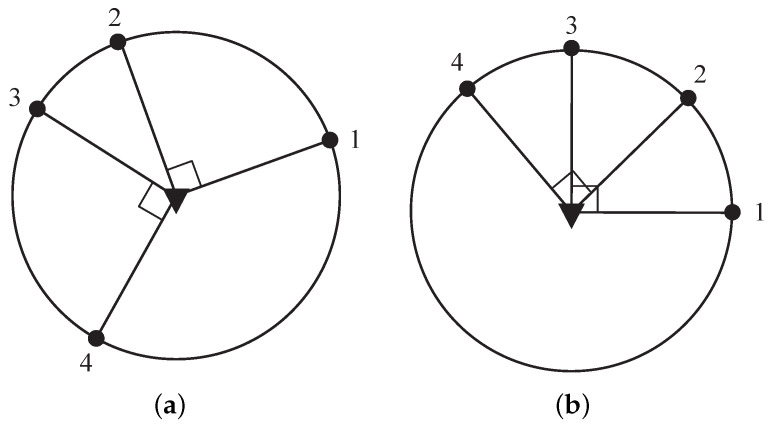
Optimal sensor–target geometries for n=4. (**a**) $S_{1}=\left\{1,2\right\}$, $S_{2}=\left\{3,4\right\}$. (**b**) $S_{1}=\left\{1,3\right\}$, $S_{2}=\left\{2,4\right\}$.

**Figure 5:**
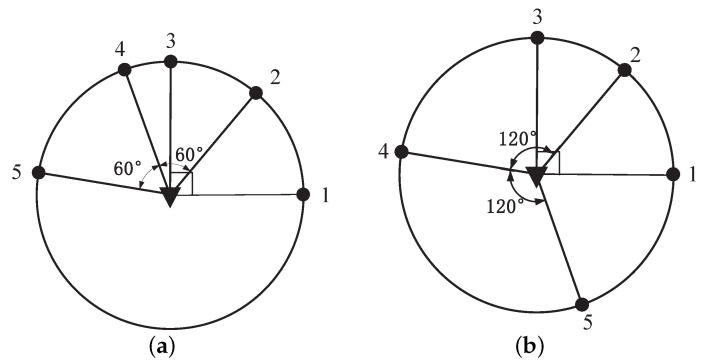

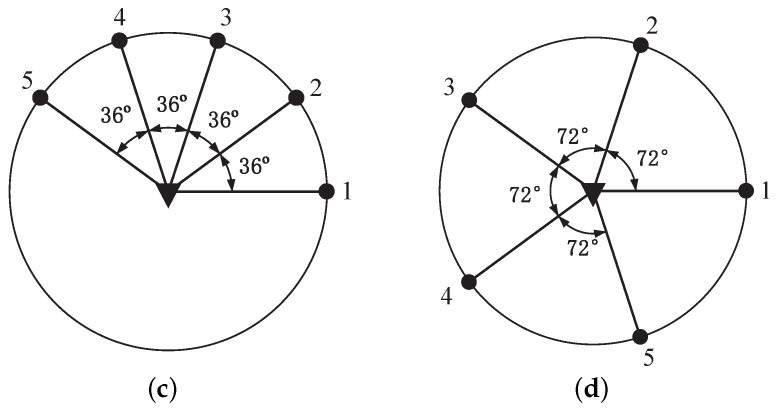
Optimal sensor–target geometry for n=5. (**a**) $S_{1}=\left\{1,3\right\},\;S_{2}=\left\{2,4,5\right\}$. (**b**) $S_{1}=\left\{1,3\right\}$, $S_{2}=\left\{2,4,5\right\}$. (**c**) $S_{1}=\left\{1,2,3,4,5\right\}$. (**d**) $S_{1}=\left\{1,2,3,4,5\right\}$.

In view of (25) in the proof of Theorem 2, the angles between the sensors not in the same subset do not affect the optimal sensor–target geometry. In addition, it remains the optimal sensor–target geometry when the sensors are managed by the geometric operations in Proposition 1. Therefore, we can classify the optimal sensor–target geometry by the set $\Xi =\{q_{1},q_{2},\ldots ,q_{m}\}$, which is recognized as the partition case dividing *n* into the sum of integers no less than 2. In other words, the optimal sensor–target geometries are regarded as identical for equivalent Ξ. Figure 4 and Figure 5 illustrate some examples of the optimal sensor–target geometry for n=4,5. In Figure 4a,b, two sensor–target geometries are considered the same because the sensors are both divided into two subsets with Ξ={2,2}. Additionally, the sensors with the same Ξ={2,3} in Figure 5a,b are also regarded as having identical sensor–target geometry, because the optimal sensor–target geometry in Figure 5b can be obtained by flipping sensor 4 about the target in Figure 5a. Additionally, the optimal sensor–target geometry with another partition case for n=5 is shown in Figure 5c,d, which is regarded as identical optimal geometry with Ξ={5,}, but they differ from the optimal geometry in Figure 5a,b due to different partition cases.

**Remark 3.**  *Although the number of optimal sensor–target geometries described in Theorem 2 is infinite due to rotation invariance, we are only concerned with the partition cases of the set S according the classification method in this paper. The number of the partition cases dividing n into a sum of positive integers no less than 2, denoted as A(n), asymptotically equals $\frac{1}{4\sqrt{3}n}\exp\left(\pi \sqrt{\frac{2n}{3}}\right)-\frac{1}{4\sqrt{3}\left(n-1\right)}\exp\left(\pi \sqrt{\frac{2(n-1)}{3}}\right)$ [37].*

## 5. Motion Coordination

**Figure 6:**
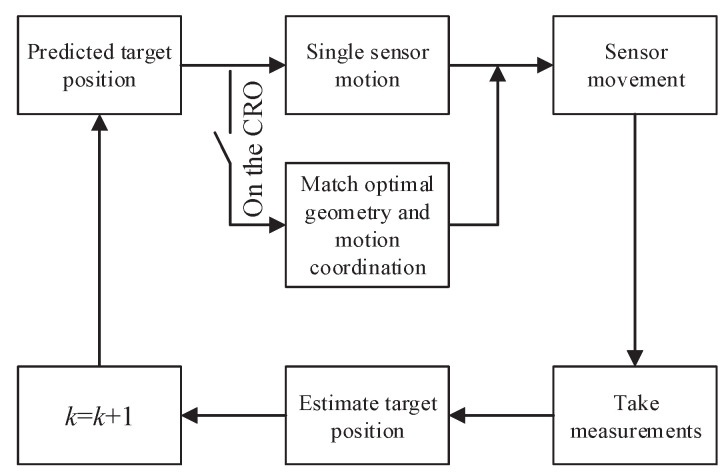
Sensor motion coordination to achieve optimal geometry.

In this section, we propose a motion coordination strategy for mobile sensors to improve target tracking performance. According to our analysis above, the mobile sensors are required to reach the CRO around the target as soon as possible and coordinate with each other. Figure 6 illustrates the main steps of sensor motion coordination to achieve optimal geometry.

### 5.1. Single Sensor Motion

In practice, the real state of the target is unknown. We utilize the one-step predicted position of the target ${\hat{p}}_{k+1|k}={\left[{\hat{x}}_{k+1|k}^{p},{\hat{y}}_{k+1|k}^{p}\right]}^{T}$ instead of $p_{k+1}$ at time *k*. The velocity of sensor *i* is designed as

(28) $$u_{i}\left(k\right)=\left\{\begin{array}{cc} v_{\max}T{\left[\cos\phi_{i,k},\sin\phi_{i,k}\right]}^{T} & r_{i,k}>d_{\min}\\ \left({\hat{p}}_{k+1|k}-{\hat{p}}_{k|k}\right) & r_{i,k}=d_{\min} \end{array}\right.$$

As we want the sensors to approach the target as soon as possible, the velocities of the sensors are set to their maximum before they reach the boundary of the CRO around the target. After the sensors reach the CRO around the target, they are expected to follow the target on the CRO around the target.

### 5.2. Coordination Strategy

As all sensors reach the CRO around the target, they enter the coordination stage. The coordination strategy consists of matching the optimal sensor–target geometry and sensor motion optimization. The task of matching the optimal geometry involves allocateing the sensors into the subsets by comparing current sensor–target geometry with optimal geometry with the desired partition case Ξ. The sensor motion is optimized to achieve the optimal geometry with minimum energy consumption based on the result of matching the optimal geometry.

Let ${\hat{s}}_{i}\left(k+1\right)={\left[{\hat{x}}_{i}\left(k+1\right),{\hat{y}}_{i}\left(k+1\right)\right]}^{T}$ denote the expected location of sensor *i* at time k+1 calculated by $u_{i}\left(k\right)$ in (28) as

(29) $${\hat{s}}_{i}\left(k+1\right)=s_{i}\left(k\right)+u_{i}\left(k\right)$$

Define ${\hat{\theta }}_{i}$ as the predicted angle

(30) $${\hat{\theta }}_{i}=\tan^{-1}\left(\frac{{\hat{y}}_{k+1|k}^{p}-{\hat{y}}_{i}\left(k+1\right)}{{\hat{x}}_{k+1|k}^{p}-{\hat{x}}_{i}\left(k+1\right)}\right)$$

where ${\hat{\theta }}_{i}$ is constrained within the range of 0 to π to simplify the step of matching the optimal geometry.

Matching optimal geometry for a given $\Xi =\{q_{1},q_{2},\ldots ,q_{m}\}$ can be described as follows:

(31) $$\begin{array}{cc} \min\; & \kappa =\sqrt{\sum_{i=1}^{m}\sum_{l=1}^{q_{i}-1}{\left({\hat{\theta }}_{n_{l+1}^{i}}-{\hat{\theta }}_{n_{1}^{i}}-\frac{l}{q_{i}}\pi \right)}^{2}}\\ s.t. & \;S_{i}\cap S_{j}=\emptyset ,\;i\neq j\\ \; & \;S_{i}=\left\{n_{1}^{i},n_{2}^{i},\ldots ,n_{q_{i}}^{i}\right\}\subset S\\ \; & \;|S_{i}|=q_{i},\;\forall i\in \left\{1,2,\ldots ,m\right\}\\ \; & \;\forall l\in \{1,2,\ldots ,q_{i}-1\} \end{array}$$

where κ is defined as the difference degree compared with the optimal sensor–target geometry. The problem is naturally a combinatorial optimization problem, which is NP-hard. An algorithm to search for an approximate solution with a given Ξ was developed and is shown in Algorithm 1 based on the greedy search method.
**Algorithm 1** Matching optimal geometry.**Input:**     S={1,2,…,n}, $\Xi =\{q_{1},q_{2},\ldots ,q_{m}\}$;**Output:**     The sensor grouping $S_{1},S_{2},\ldots ,S_{m}$;1:**for** i=1,…,m **do**2:   **for** j∈S **do**3:     **for** $l=2,\ldots ,q_{i}$ **do**4:                 $L_{j}^{l}=\underset{k\in S\backslash \{j\}}{\arg\min}{\left({\hat{\theta }}_{k}-{\hat{\theta }}_{j}-\frac{l-1}{q_{i}}\pi \right)}^{2}$;5:     **end for**6:           $\kappa_{j}=\sqrt{\sum_{l=2}^{q_{i}}{\left({\hat{\theta }}_{L_{j}^{l}}-\hat{\theta_{j}}-\frac{l-1}{q_{i}}\pi \right)}^{2}}$;7:   **end for**8:   Find the minimum $\kappa_{j}$, $S_{i}⟵\left\{j,L_{j}^{2},\ldots ,L_{j}^{q_{i}}\right\}$,       $S⟵S\backslash S_{i}$;9:**end for**10:**return**$\left\{S_{1},S_{2},\ldots ,S_{m}\right\}$.

**Remark 4.**  *The step of matching the optimal geometry only needs to be performed once when the sensors all reach the CRO. The sensor coordination follows the optimal geometry matched via Algorithm 1 in later sensor movement on the CRO. Moreover, the computation complexity of Algorithm 1 is $O\left(mn^{2}\right)$.*

After matching the optimal sensor–target geometry, the sensors engage in motion coordination to achieve the optimal geometry, thereby improving tracking performance. For the purpose of energy conservation, sensor motion optimization can be described as a nonlinear optimization problem

(32) $$\begin{array}{cc} \min\; & \vartheta =\sum_{i=1}^{m}\sum_{j=1}^{q_{i}-1}∥{\bar{u}}_{n_{j}^{i}}\left(k\right)∥\\ s.t.\; & {\hat{\theta }}_{n_{j+1}^{i}}^{*}-{\hat{\theta }}_{n_{1}^{i}}^{*}=\frac{j}{q_{i}}\pi \\ \; & ∥s_{n_{j}^{i}}^{*}\left(k+1\right)-{\hat{p}}_{k+1|k}∥=d_{\min}\\ \; & j\in \left\{1,2,\ldots ,q_{i}-1\right\},\;i\in \left\{1,2,\ldots ,m\right\} \end{array}$$

where ϑ is the sum of the distance traveled by the sensors; ${\bar{u}}_{n_{j}^{i}}\left(k\right)=s_{n_{j}^{i}}^{*}\left(k+1\right)-{\hat{s}}_{n_{j}^{i}}\left(k+1\right)$ and ${\hat{\theta }}_{i}^{*}$ are the predicted angles for $s_{i}^{*}\left(k+1\right)$. The nonlinear optimization problem in (32) can be solved by “fmincon” (Optimization toolbox) in Matlab®. Therefore, the control input for the sensor *i* is finally determined by

(33) $$\begin{array}{cc} u_{i}\left(k\right) & =u_{i}\left(k\right)+{\bar{u}}_{i}\left(k\right)\\ \; & =s_{i}^{*}\left(k+1\right)-s_{i}\left(k\right) \end{array}$$

The restriction of the turn rate can be implemented by choosing $\min\{\varphi_{\max},|∠u_{i}\left(k\right)-∠u_{i}\left(k-1\right)|\}$.

**Remark 5.**  *In terms of bearings-only target tracking accuracy, both the enveloping and semienveloping optimal sensor–target geometry configurations are considered equivalent. The selection of the configurations depends on the objectives of target tracking. When the sensors are expected to perform other operations, such as surveillance, recording, and so on, circumnavigation tracking is a more preferable approach, driving the sensors to achieve complete surrounding of a target on the CRO.*

### 5.3. Collision Avoidance

A distance constraint is necessary to avoid collisions among the mobile sensors. Let $l_{\min}$ denote the minimum distance between two sensors. When $∥s_{i}\left(k\right)-s_{j}\left(k\right)∥<\rho_{\min}$, the collision avoidance algorithm is enabled, and we have

(34) $$\begin{array}{cc} s_{i}\left(k+1\right) & =\left[\begin{array}{c} x_{i}\left(k\right)+∥u_{i}\left(k\right)∥\cos\left(∠u_{i}\left(k\right)\pm \delta \right)\\ y_{i}\left(k\right)+∥u_{i}\left(k\right)∥\sin\left(∠u_{i}\left(k\right)\pm \delta \right) \end{array}\right]\\ s_{j}\left(k+1\right) & =\left[\begin{array}{c} x_{j}\left(k\right)+∥u_{j}\left(k\right)∥\cos\left(∠u_{j}\left(k\right)\pm \delta \right)\\ y_{j}\left(k\right)+∥u_{j}\left(k\right)∥\sin\left(∠u_{j}\left(k\right)\pm \delta \right) \end{array}\right] \end{array}$$

where δ is a small heading change for the sensor, and ±δ is selected to make the range between them larger.

To summarize, the sensor motion coordination algorithm is presented in the Algorithm 2.
**Algorithm 2** Sensor motion coordination for target tracking.**Input:**     The estimate of the target at time *k*, ${\hat{x}}_{k|k}$;  The location of the sensor at time *k*, $s_{i}\left(k\right)$;**Output:**     The estimate of the target at time k+1, ${\hat{x}}_{k+1|k+1}$;  The location of the sensor at time k+1, $s_{i}\left(k+1\right)$;1:Receive ${\hat{x}}_{k+1|k}$ from the estimation center;2:Compute $u_{i}\left(k\right)$ with (28), (31), (33) and (34);3:Move to a new position $s_{i}\left(k+1\right)$;4:Take new measurements of the target $z_{k+1}$, and estimate the state of the target via CKF;5:**return** ${\hat{x}}_{k+1|k+1}$, $s_{i}\left(k+1\right)$.

## 6. Simulation Experiments

In this section, we illustrate the proposed sensor motion coordination algorithm with some simulation examples. By default, all variables used in the simulation were in SI units. As introduced in Section 3.1, we used a CKF method to estimate the state of the target. For comparison, the gradient descent method in [34] and the projection method in [35] were adopted to optimize the sensor motion under the same conditions.

To compare the tracking performance, we used the root mean square error (RMSE) of the position of the target. The RMSE of position at time *k* is defined as

$${\mathrm{RMSE}}_{p}\left(k\right)=\sqrt{\frac{1}{N_{c}}\sum_{i=1}^{N_{c}}\left({\left(x^{i}\left(k\right)-{\hat{x}}^{i}\left(k\right)\right)}^{2}+{\left(y^{i}\left(k\right)-{\hat{y}}^{i}\left(k\right)\right)}^{2}\right)}$$

where $N_{c}$ is the total numbers of Monte Carlo runs; ${\left[x^{i}\left(k\right),y^{i}\left(k\right)\right]}^{T}$ and ${\left[{\hat{x}}^{i}\left(k\right),{\hat{y}}^{i}\left(k\right)\right]}^{T}$ are the true and estimated positions at the *n*th Monte Carlo run respectively.

Scenario 1: We consider a problem of tracking a moving target using 5 mobile sensors in 2D space. The dynamic function of the target is described by the constant velocity model

$$x_{k+1}=\left[\begin{array}{cccc} 1 & T & 0 & 0\\ 0 & 1 & 0 & 0\\ 0 & 0 & 1 & T\\ 0 & 0 & 0 & 1 \end{array}\right]x_{k}+w_{k}$$

where $x_{k}={\left[x_{k},{\dot{x}}_{k},y_{k},{\dot{y}}_{k}\right]}^{T}$ and T=0.2s is the sampling time. The process noise $w_{k}$ is a zero-mean Gaussian with a covariance matrix $Q_{k}=\mathrm{diag}\left[qM\;qM\right]$, where

$$M=\left[\begin{array}{cc} T^{3}/3 & T^{2}/2\\ T^{2}/2 & T \end{array}\right]$$

The scalar parameter $q=0.1\;m/s^{3}$ denotes the process noise intensity. The measurements taken by sensor *i* at time *k* is given in (1) and $\sigma_{\theta }=0.1\;\mathrm{rad}$.

The true initial state of the target is $x_{0}={\left[-50\;m\;3\;m/s\;50\;m\;1\;m/s\right]}^{T}$, and its associated covariance is $P_{0|0}=\mathrm{diag}\left[1000\;m^{2}\;100\;m^{2}/s^{2}\;1000\;m^{2}\;100\;m^{2}/s^{2}\right]$. The initial state estimate $x_{0|0}$ is randomly chosen from $N(x_{0},P_{0|0})$ in each run. The initial positions of the 5 sensors are $s_{1}\left(0\right)={\left[-100\;m\;120\;m\right]}^{T}$, $s_{2}\left(0\right)={\left[-150\;m\;50\;m\right]}^{T}$, $s_{3}\left(0\right)={\left[-100\;m\;60\;m\right]}^{T}$, $s_{4}\left(0\right)={\left[-100\;m\;-120\;m\right]}^{T}$, and $s_{5}\left(0\right)={\left[100\;m\;-200\;m\right]}^{T}$. The maximum velocity and turn rate are $v_{\max}=10\;m/s$ and $\varphi_{\max}=\frac{\pi }{3}\;\mathrm{rad}$, respectively. The minimum restriction is $d_{\min}=50\;m$, and the minimum distance among the sensors is $\rho_{\min}=10\;m$. Set $N_{c}=2000$.

**Figure 7:**
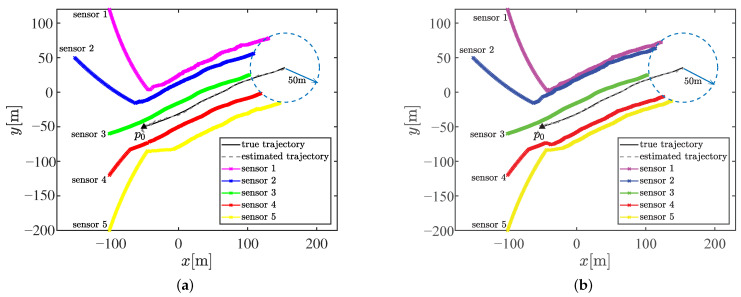
Sensor trajectory for target tracking in Scenario 1. (**a**) The optimal geometry with partition case Ξ={5}. (**b**) The optimal geometry with partition case Ξ={2,3}.

**Figure 8:**
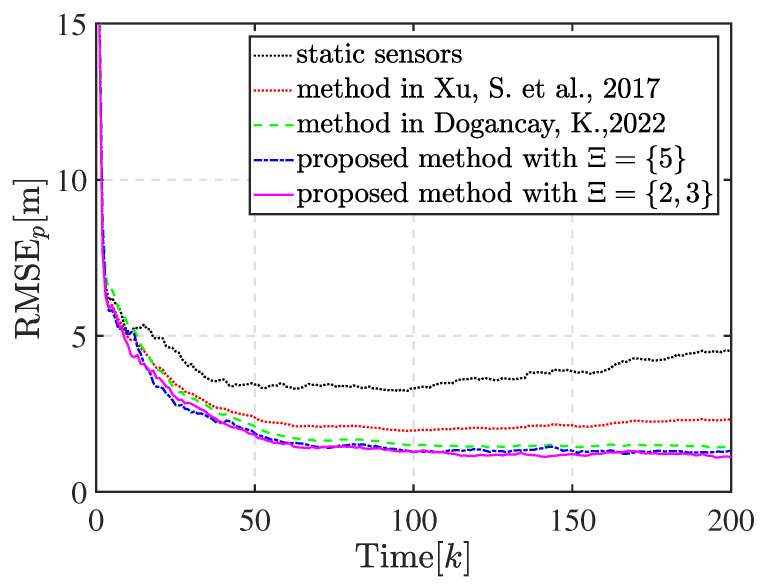
Comparison of ${\mathrm{RMSE}}_{p}$ for target tracking in Scenario 1 [34,35].

There are two partition cases for n=5 with Ξ={2,3} and Ξ={5}. We first compared the tracking performance and distance traveled by the mobile sensors when the sensors are steered to achieve these two kinds of optimal sensor–target geometries. Additionally, we included static sensors and mobile sensors whose waypoints were computed by the methods in [34,35] in the comparative experiment. Figure 7a,b show the trajectory of the 5 bearings-only sensors achieving the optimal geometry with partition cases Ξ={5} and Ξ={2,3}, respectively. As shown in Figure 7b, sensor 1, sensor 3, and sensor 5 are assigned into the subset with three sensors and the others in the subset with two sensors after matching the optimal geometry. The sensors eventually move with the target in the optimal geometry, as expected. The optimal geometry is referenced to the estimated target position and shows discrepancies with the true optimal sensor–target geometry. This discrepancy is unavoidable in practical applications since the true target position is unknown. However, the proposed motion coordination method can enhance the estimation performance, and the circular formation approaches closer to the true optimal geometry, thus achieving the theoretically optimal estimation accuracy, as shown by the compared RMSEs of the position illustrated in Figure 8. Obviously, the tracking performance estimated by mobile sensors is better than that estimated by static sensors. The proposed method significantly improves the tracking performance and exhibits lower estimate error compared with the method in [34]. Meanwhile, the tracking performance of the method in [35] is close to the proposed method in this scenario. There is a negligible difference in the tracking performance between the two kinds of optimal geometries with Ξ={2,3} and Ξ={5}. Additionally, the sums of the distance traveled by all mobile sensors to achieve the optimal geometry with Ξ={2,3} and Ξ={5} are 1488.9m and 1548.6m, respectively. The reduction in distance between Ξ={2,3} and Ξ={5} is attributed to the fact that the sensor–target geometry is closer to the optimal geometry with Ξ={2,3}, whose κ is smaller, when the sensors reach the CRO.

Scenario 2: We consider a problem of tracking a moving target using 4 mobile sensors in 2D space. The dynamic function of the target is described by

$$x_{k+1}=\left[\begin{array}{ccccc} 1 & \frac{\sin\Omega_{k}T}{\Omega_{k}} & 0 & -\left(\frac{1-\cos\Omega_{k}T}{\Omega_{k}}\right) & 0\\ 0 & \cos\Omega_{k}T & 0 & -\sin\Omega_{k}T & 0\\ 0 & \frac{1-\cos\Omega_{k}T}{\Omega_{k}} & 1 & \frac{\sin\Omega_{k}T}{\Omega_{k}} & 0\\ 0 & \sin\Omega_{k}T & 0 & \cos\Omega_{k}T & 0\\ 0 & 0 & 0 & 0 & 1 \end{array}\right]x_{k}+w_{k}$$

where $x_{k}={\left[x_{k},{\dot{x}}_{k},y_{k},{\dot{y}}_{k},\Omega_{k}\right]}^{T}$ and T=1s. The process noise $w_{k}$ is a zero-mean Gaussian with a covariance matrix $Q_{k}=\mathrm{diag}\left[q_{1}\Gamma \;q_{1}\Gamma \;q_{2}T\right]$, where

$$\Gamma =\left[\begin{array}{cc} T^{3}/3 & T^{2}/2\\ T^{2}/2 & T \end{array}\right]$$

and $q_{1}=0.1\;m/s^{3}$ and $q_{2}=1.75\times 10^{-4}\;\mathrm{rad}/s^{2}$ denote the process noise intensity. The true initial state of the target is $x_{0}={\left[0\;m\;20\;m\;0\;m\;0\;m\;0.05\;\mathrm{rad}/s\right]}^{T}$, and its associated covariance is $P_{0|0}=\mathrm{diag}\left[1000\;m^{2}\;100\;m^{2}/s^{2}\;1000\;m^{2}\;100\;m^{2}/s^{2}\;10^{-4}\;{\mathrm{rad}}^{2}/s^{2}\right]$. The initial positions of the 4 sensors are randomly deployed. The rest parameters are listed as: $\sigma_{\theta }=0.05\;\mathrm{rad}$, $d_{\min}=100\;m$, $\rho_{\min}=20\;m$, $v_{\max}=50\;m/s$, $\varphi_{\max}=\frac{\pi }{3}\;\mathrm{rad}$ and $N_{c}=2000$.

**Figure 9:**
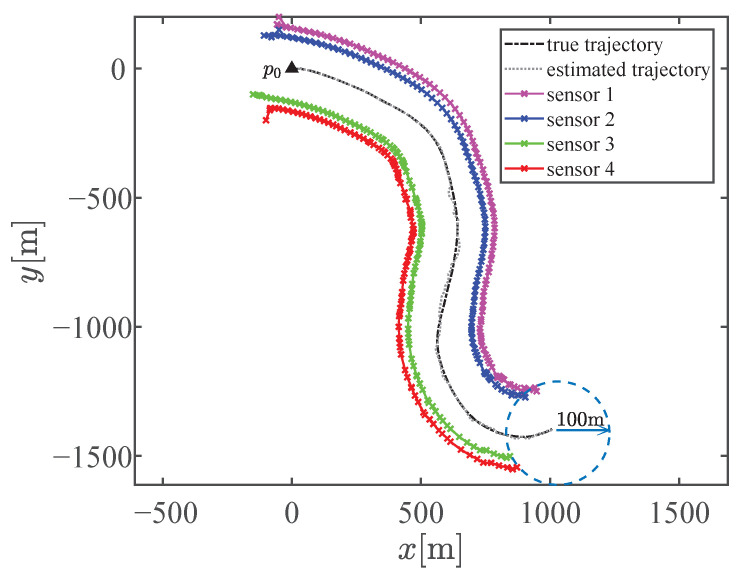
Sensor trajectory for target tracking in Scenario 2.

**Figure 10:**
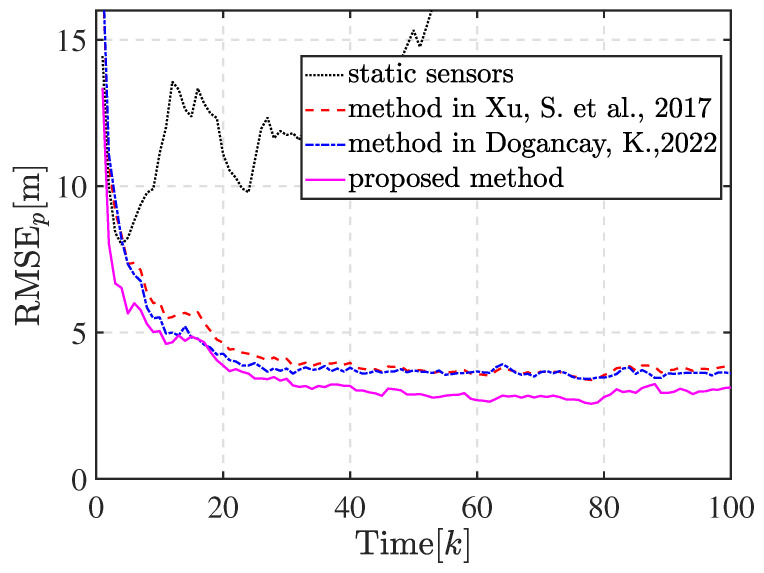
Comparison of ${\mathrm{RMSE}}_{p}$ for target tracking in Scenario 2 [34,35].

There are two partition cases for n=4 with Ξ={2,2} and Ξ={4}. However, the optimal geometry with Ξ={4} can be obtained by rotating the sensors in one subset in the optimal geometry with Ξ={2,2} as a whole by a proper angle. Thus, the partition case for n=4 is selected as Ξ={2,2} in Scenario 2. Figure 9 shows the trajectory of the 4 bearings-only sensors tracking a target. In this run, sensors 1 and 3 are assigned in a subset and the others in another subset after matching the optimal geometry. Figure 10 shows the compared RMSEs of the position. Obviously, the tracking performance estimated by static sensors is the poorest, and it continues to degrade as the distance from the target increases. The proposed method improves the tracking performance and exhibits lower estimate error compared with the methods in [34,35] for maneuver turning target tracking.

## 7. Conclusions

In this study, optimal sensor–target geometry and a motion coordination strategy were proposed for a target tracking system using mobile bearings-only sensors in 2D space. We discussed the suboptimality of approaching the target for bearings-only sensors to improve tracking performance. A general optimal sensor–target geometry was derived with uniform sensor–target distance using D-optimality for arbitrary *n* (n≥2) bearings-only sensors. A motion coordination algorithm was developed based on the previous optimality analysis to achieve the optimal target tracking performance efficiently. In future work, we will investigate a distributed optimization method for mobile sensors and its extension to multitarget tracking.

## Data Availability

Not applicable.
